# Two Rhabdoviruses, One Novel, Isolated from *Armigeres subalbatus* in China

**DOI:** 10.3390/pathogens11060624

**Published:** 2022-05-27

**Authors:** Xiuyan Xu, Jing Wang, Hong Liu, Qinyan Wang, Shihong Fu, Jun Zhang, Bin Wang, Ying He, Fan Li, Kai Nie, Songtao Xu, Huanyu Wang, Xiaoqing Lu, Mang Shi, Guodong Liang

**Affiliations:** 1 Beijing Center for Disease Prevention and Control, Beijing 100013, China; xuxiuyan@bjcdc.org; 2 State Key Laboratory of Infectious Disease Prevention and Control, National Institute for Viral Disease Control and Prevention, Chinese Center for Disease Control and Prevention, Beijing 102206, China; wqy2738781242@126.com (Q.W.); fush@ivdc.chinacdc.cn (S.F.); heying@ivdc.chinacdc.cn (Y.H.); lifan@ivdc.chinacdc.cn (F.L.); niekai@ivdc.chinacdc.cn (K.N.); xust@ivdc.chinacdc.cn (S.X.); 3 School of Public Health, Qingdao University, Qingdao 266071, China; wangbin532@126.com; 4 School of Medicine, Sun Yat-sen University, Shenzhen 518107, China; wangj796@mail2.sysu.edu.cn; 5 School of Biomedicine, Shandong University of Technology, Zibo 255000, China; liuhongseminar@sdut.edu.cn (H.L.); zhangj@qlmu.edu.cn (J.Z.)

**Keywords:** *almendravirus*, *Armigeres subalbatus*, rhabdovirus, Shanxi Arboretum virus (SXABTV), Shanxi *Armigeres subalbatus* rhabdovirus (SXARV)

## Abstract

The family *Rhabdoviridae* contain important human and mammalian pathogens that are vectored by different arthropod species. The ground supernatants of mosquitoes were used to inoculate in BHK-21 and C6/36 cells for virus isolation. Then, the viral complete genome sequence was obtained and used for phylogenetic analysis. In this study, we observed a cytopathic effect (CPE) in mosquito cells (C6/36) and rod-like virion after inoculating a pool of *Armigeres subalbatus* samples collected in Shanxi Province, China, in 2019 (SX1916). Meta-transcriptomics sequencing revealed the presence of two distinctive rhabdoviruses with similar abundance levels, namely, Shanxi *Armigeres subalbatus* rhabdovirus (SXARV) and Shanxi Arboretum virus (SXABTV). Despite the fact that the SXARV genome (9590 nt) was much shorter than that of SXABTV (11,480 nt), both belonged to the *Almendravirus* group within *Rhabdoviridae* whose genomes encoded five proteins (N, P, M, G, and L) and a small hydrophobin (U1) and the difference in lengths is mainly caused by a substantially shorter N protein encoded by SXARV. On the phylogenetic tree, SXABTV was closely related (90.7% amino acid identity at L protein) with the Arboretum virus isolated from Psorophora albigenu mosquitoes in Peru in 2014, whereas SXARV was distantly related to Rio Chico virus (63.3% amino acid identity), a genetic distance large enough to be defined as a new species within *Rhabdoviridae*. Collectively, we report a simultaneous isolation of two related rhabdoviruses from *Armigeres subalbatus* that marked the circulation of almendraviruses in Shanxi, China.

## 1. Introduction

Rhabdovirus is a single-stranded negative-sense RNA virus that is rod or bullet shaped, with a particle length of 100–430 nm and diameter of 45–100 nm. According to the latest virological classification report from the International Committee on Taxonomy of Viruses (ICTV), the family *Rhabdoviridae* comprises 45 genera and 275 species [1]. *Rhabdoviridae* viruses have a wide host range, including arthropods, such as mosquitoes, ticks, sandflies, birds, fish, and mammals [2,3,4].

*Almendravirus* (*Rhabdoviridae*) virus had a full-length genome of approximately 11,000 nt, which mainly encode five proteins, namely, nucleoprotein (N), phosphoprotein (P), and matrix protein (M), glycoprotein (G), RNA-dependent RNA polymerase protein (L), as well as a small hydrophobin (U1) between the G and L genes, following the order 3′N-P-M-G-U1-L5′ [4]. The U1 protein in the viral genome has unique characteristics that are used to identify *Almendravirus* viruses [5], which have been isolated in North America, South America, and Asia [5,6,7].

To date, the ICTV has established seven viral species within the genus *Almendravirus*: Arboretum virus and Puerto Almendras viruse isolated from Peru [7], Balsa virus isolated from Colombia [5], Coot Bay virus isolated from USA [5], Menghai rhabdovirus isolated from China [6], and Japan Rhabdovirus [8] and Rio Chico virus isolated from Panama [5]. Among these, only Menghai rhabdovirus was extensively described in China, including a strain isolated from Aedes albopictus collected in 2010 [6] and five strains isolated from midges collected in 2018. In addition, *Almendravirus* has not been isolated from bloodsucking insects collected in China.

From June to August 2019, we conducted comprehensive sampling of different species of vectors, including sandfly and mosquito in Wuxiang County, Shanxi Province, Central China. A number of viruses have been identified from these vectors, including Wuxiang virus isolated from sandfly samples [9]. In this study, we report two virus strains isolated from *Armigeres subalbatus*, both belonging to the *Almendravirus* genus (*Rhabdoviridae*).

## 2. Results

### 2.1. Collection of Mosquito Specimens

A total of 782 mosquitoes from four genera and four species was collected in this study (Table S1), including 340 *Anopheles sinensis*, 360 *Armigeres subalbatus*, 37 *Aedimorphus vexans*, and 45 *Culex pipiens pallens*. *Anopheles sinensis* and *Armigeres subalbatus* accounted for 43.48% (340/782) and 46.04% (360/782), respectively, of total mosquito specimens collected and are, therefore, considered the dominant species in Wuxiang County.

### 2.2. Virus Isolation and Preliminary Identification

**Figure 1:**
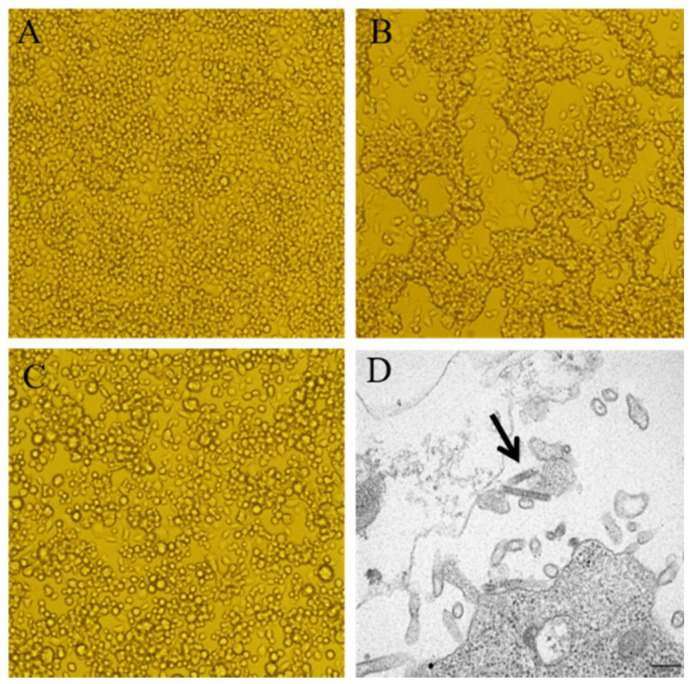
C6/36 cell cytopathic effect (CPE) caused by virus isolates and electron microscopic morphology of the virus. (**A**) Normal C6/36 cell morphology on day 4; (**B**) CPE in fourth-generation C6/36 cells inoculated with the SX1916 isolate on day 4, showing the aggregation of large numbers of cells, but no cell shedding; (**C**) CPE in second-generation C6/36 cells inoculated with plaque-purified SXARV (SXARV1916-2) on day 4, showing large numbers of fused cells and cell shedding; (**D**) SXARV morphology examined by electron microscopy. Arrow indicates a rod-shaped virus particle. Magnification for (**A**–**C**): ×200.

The collected mosquito specimens were pooled into 16 samples and homogenized. Each pool contains 30~50 mosquitoes and there are a total of seven pools for *Anopheles sinensis*, seven for *Armigeres subalbatus*, one for *Aedimorphus vexans,* and one for *Culex pipiens pallens*. The resulting supernatant was inoculated onto BHK-21 or C6/36 cells and the cells were cultured continuously and observed daily for CPE. For the pooled *Armigeres subalbatus* sample SX1916, the first passage in C6/36 cells showed no CPE. CPE appeared in the second generation on day 4, characterized by the aggregation of large numbers of cells and cell space enlargement (Figure 1B). The cell supernatant showing CPE was re-inoculated onto C6/36 cells for passages 3 and 4, and CPE again appeared on day 4, suggesting that CPE caused by SX1916 can be stably passaged. On the other hand, the supernatant from SX1916 isolate was inoculated onto BHK-21 cells for three consecutive generations and no CPE was observed. To identify the infectious agent that causes CPE, we first used multiple arbovirus group/genera-specific gene amplification primers (flavivirus, alphavirus, and bunyavirus) and virus species-specific gene amplification primers (Japanese encephalitis virus, Banna virus, Tibet orbivirus, Densovirus, and Totivirus) for gene amplification testing via reverse-transcription (RT)-PCR of the SX1916 virus isolate (Table S2). None of the primers used resulted in positive results.

**Figure 2:**
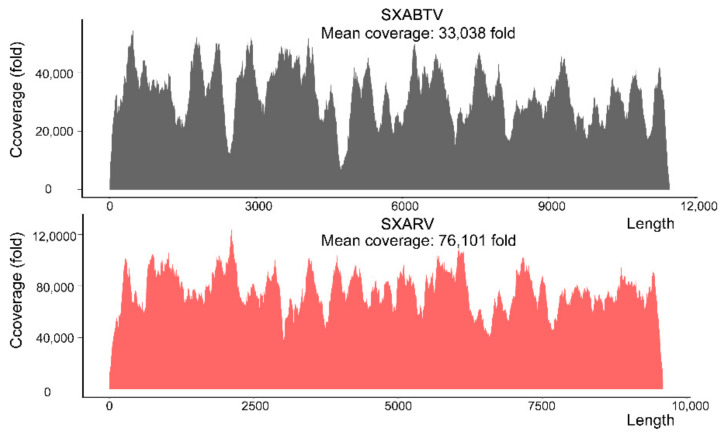
Coverage and sequencing depth of the SXABTV and SXARV genome.

To identify the viruses from the SX1916 isolate, we extracted the total RNA of the supernatant of C6/36 cells infected with the SX1916 isolate for next-generation sequencing (NGS). The sequencing resulted in 9,144,290 reads, which were assembled into 4969 contigs. Taxonomy annotation of all contigs revealed two rhabdovirus genome sequences in the SX1916 isolate, with lengths of 11,480 and 9590 nt, respectively. These viruses were tentatively named as Shanxi Arboretum virus (SXABTV, strain SXABTV1916-1) and Shanxi *Armigeres subalbatus* rhabdovirus (SXARV, strain SXARV1916-2). Importantly, both rhabdoviruses had very high mean coverage, namely, 33,038-fold for SXABTV and 76,101-fold for SXARV (Figure 2), which account for 28.5% and 5.6% of total RNA sequenced, suggesting the two viruses are highly abundant in the cell supernatant. In addition, we searched for other potential virus contigs from our annotation results (Table S3). However, other virus sequences contain only Guadeloupe mosquito quaranja-like virus 3 (423 bp) and Guato virus (474 bp) and they are too short for analysis.

### 2.3. Virus Plaque Purification

Because the NGS results identified two rhabdoviruses with large genomic differences in the SX1916 isolate, we applied virus plaque purification methods to purify each rhabdovirus. The SX1916 isolate was used for four consecutive passages of the fourth-passage C6/36 cells for virus plaque purification. In the first and second plaque purification, the virus-infected C6/36 cells were cultured for 7 days (a short-diameter plaque was observed on day 6) to obtain a single virus plaque. We obtained nine and three single virus plaques in the first and second purification tests, respectively (Figure S1). All 12 plaques had similar diameters (mean, 2.4 ± 0.5 mm; *n* = 12); they were thawed and diluted with cell culture medium to extract RNA and subjected to gene detection. The results of parallel detection of SXARV and SXABTV genes showed that all 12 plaques were SXARV gene positive and SXABTV gene negative. Plaques that tested positive for SXARV were diluted with cell culture medium and subjected to additional plaque purification. After three rounds of plaque purification, an SXARV isolate (SXARV1916-2) was obtained. All subsequent studies involving SXARV came from the SXARV1916-2 strain.

To confirm that sample used before the plaque purification test contained both viruses, we performed parallel genetic testing of both viruses in fourth-generation C6/36 cells inoculated with the SX1916 isolate to verify whether both viruses were present in the cell supernatant used in the plaque purification test. The results showed simultaneous amplification of the viral gene amplification products of both SXARV and SXABTV in the fourth-generation supernatant of the C6/36 cells (Figure S2). 

The plaque-purified SXARV1916-2 isolate was inoculated into C6/36 cells to observe its cytopathic characteristics. In second-generation C6/36 cells inoculated with plaque-purified SXARV1916-2, CPE was clearly observed after 4 days of culture, manifesting as large numbers of fused cells and cell shedding (Figure 1C). However, in fourth-generation C6/36 cells inoculated with the SX1916 virus isolate, CPE on day 4 was characterized by the aggregation of large numbers of cells, but not cell shedding (Figure 1B). Thus, CPE differed significantly between C6/36 cells inoculated with purified SXARV1916-2 and those inoculated with the SX1916 virus isolate containing two virus strains.

### 2.4. Electron Microscopy of Virus Morphology

Plaque-purified SXARV was inoculated into C6/36 cells and ultra-thin section detection was performed when the cells showed clear CPE. Rod-shaped virus particles (length, 300 nm; diameter, 70 nm) were observed via electron microscopy (Figure 1D and Figure S3).

### 2.5. Molecular Biological Characteristics of the SXARV and SXABTV Isolates

#### 2.5.1. Nucleotide Sequence of the Viral Genome Coding Region

According to the virus genome sequence information obtained via NGS sequencing, we designed amplification primers to cover the full-length genome nucleotide sequence of SXARV and SXABTV, respectively, using Primer-BLAST (https://www.ncbi.nlm.nih.gov/tools/primer-blast (accessed on 5 April 2021)), and the primers used are listed in Table S4. The cDNA library of the fourth-generation supernatant of C6/36 cells inoculated with the SX1916 isolate was used as a template. PCR amplification and nucleotide sequence determination of the whole genome of each rhabdovirus were conducted using the designed primers. The non-infected control C6/36 cell lines were negative for both viruses discovered in this study. The obtained whole-genome nucleotide sequences of SXABTV and SXARV were uploaded to GenBank under accession nos. MW890015 and MW890016, respectively.

**Figure 3:**
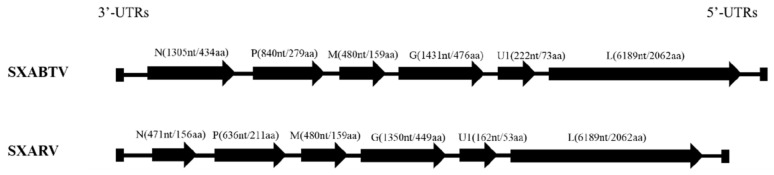
Genome structures of the SXABTV and SXARV isolates, both of which encode N, P, M, G, U1, and L proteins in the 3′–5′ order.

Sequence analyses of SXABTV and SXARV genomes showed that both viruses encode six proteins: N proteins of 1305 nt/434 aa and 471 nt/156 aa, P proteins of 840 nt/279 aa and 636 nt/211 aa, M proteins of 480 nt/159 aa and 480 nt/159 aa, G proteins of 1431 nt/476 aa and 1350 nt/449 aa, and L proteins of 6189 nt/2062 aa and 6189 nt/2062 aa. The viral genomes of SXABTV and SXARV also contained genes U1 (222 nt/73 aa and 162 nt/53 aa) between the G and L proteins of the small hydrophobin (Figure 3). The difference in length between SXABTV and SXARV is mainly caused by a substantially shorter N protein encoded by SXARV.

#### 2.5.2. Nucleotide and Amino Acid Sequence Identity in Viral Gene Coding Regions

We examined the identity of nucleotides and amino acids in the coding regions of SXABTV and SXARV and other *Almendravirus* viruses, including 13 isolate strains in GenBank, and found that SXABTV and Arboretum virus (ABTV) had the highest identity. We identified nucleotide (amino acid) identity of the coding region of the N gene at 83.9% (97.5%), the P gene at 70.8% (68.5%), the M gene at 79.3% (88.5%), the G gene at 75.6% (84.1%), the U1 gene at 69.4% (52.9%), and the L gene at 78.9% (90.7%).

Nucleotide and amino acid identity of the coding regions of the SXARV isolate had the highest identity with the Rio Chico virus. We identified nucleotide (amino acid) identity of the coding region of the N gene at 59% (52.8%), the P gene at 55% (54.8%), the M gene at 59.5% (48.1%), the G gene at 53.4% (41.7%), the U1 gene at 57.5% (49.4%), and the L gene at 64.6% (63.3%) (Table 1).

The virus nucleotide and amino acid identity data (Table 1) indicate that *Almendravirus* contains seven virus species: ABTV, Balsa virus, Menghai rhabdovirus virus, Rio Chico virus, SXARV, Coot Bay virus, and Puerto Almendras virus. Recent studies have reported that amino acid identity of the coding region of the L protein of <90% is sufficient for identifying new rhabdovirus species. The amino acid identity of the L gene coding region varies among the seven viruses from 40% (ABTV and Menghai rhabdovirus virus, kunoichi strains GD18003 and GD18005) to 64.1% (ABTV and Puerto Almendras virus).

#### 2.5.3. Evolutionary History of SXABTV and SXARV

**Figure 4:**
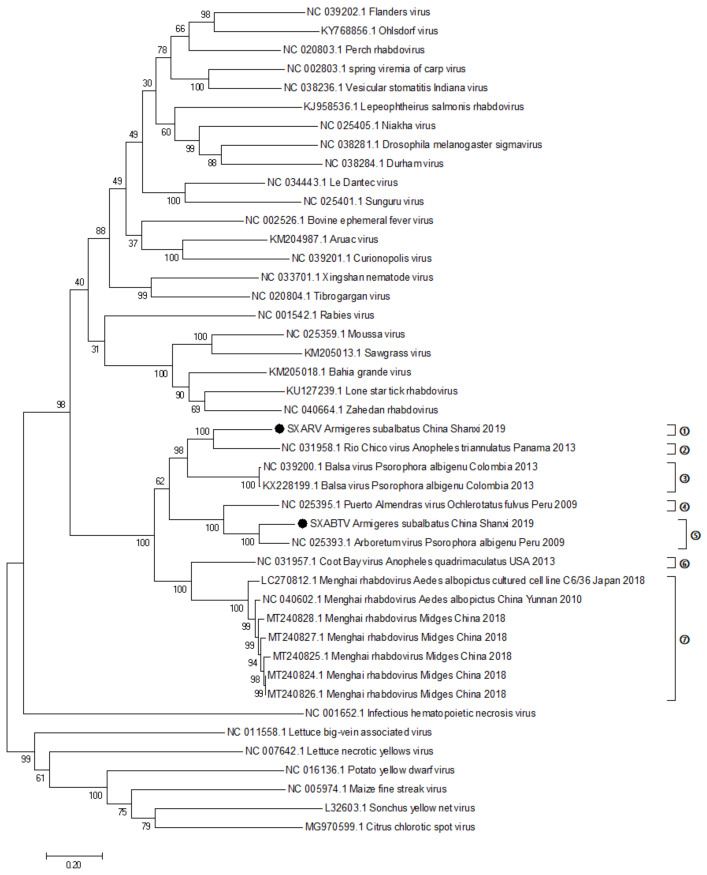
Evolution of the L gene coding regions of SXABV and SXARV based on nucleotide sequences. Black marks indicate the two rhabdoviruses isolated in this study. Systematic evolution analysis based on the maximum likelihood method, with a bootstrap value of 1000. Numbers 1–7 represent seven viruses in genus *Almendravirus*. The scale bar represents the unit length of the value of the difference between sequences.

To analyze the phylogenetic relationship between the SXABTV and SXARV and other *Almendravirus* viruses, we prepared an analysis dataset of the L gene sequence of *Rhabdoviridae* viruses, including all *Almendravirus* viruses and representative strains of 29 other genera of *Rhabdoviridae* (Table S5). Phylogenetic analysis results obtained using the maximum likelihood method showed that both SXABTV and SXARV were in the *Almendravirus* genus, and that SXABTV was closely related to an ABTV isolate obtained from mosquitoes collected in Peru, whereas SXARV was closely related to a Rio Chico virus isolate obtained from mosquitoes collected in Panama (Figure 4).

## 3. Discussion

In this study, we obtained virus isolate SX1916 from *Armigeres subalbatus* collected in Shanxi Province in Central China in 2019. SX1916 was found to cause CPE in C6/36 cells and was stably passaged (Figure S5). Virology and molecular biology analyses showed that SX1916 contained two rhabdoviruses, SXABTV and SXARV.

Plaque purification can be used to clone virus isolates or variants with different morphological characteristics, including plaque diameter [10]. We applied this technique to clone separate isolates of SXARV and SXABTV from the SX1916 isolate. However, all of the plaques obtained in two plaque purification assays belonged to SXARV, and no SXABTV plaques were obtained, which suggested that SXABTV may not be capable of forming plaques. A study on the plaque-forming ability of 212 arboviruses (including serotypes) in Vero and LLC-MK2 cells showed that 169 arboviruses can form plaques in these two cell lines, whereas 17 and 18 arboviruses could not form plaques in VERO and LLC-MK2 cells, respectively, and the remaining seven could not form plaques in either cell line [11]. The results of that study suggested that some important mosquito-borne viruses, such as Zika virus, can cause CPE, but not form plaques, in Vero cells [12]. Thus, not all arboviruses have the ability to form plaques. ABTV can cause CPE in C6/36 cells, but whether it can form plaques in this cell line has not been reported [7]. The SXABTV isolate failed to form plaques in C6/36 cells; however, further research is required to conclude that it has no plaque-forming properties. It is possible that the presence of SXARV inhibited plaque forming by SXABTV in the present study. Interference between the two rhabdoviruses may also have affected their cytopathic properties. After the SX1916 isolate was inoculated with C6/36 cells, the cells showed significant aggregation, but not cell shedding. However, after plaque-purified SXARV was inoculated onto C6/36 cells, these cells showed substantial cell fusion and shedding. These differences may be caused by mutual interference between the co-infecting SXABTV and SXARV, which was then eliminated in plaque-purified SXARV. A study of snakehead retrovirus (Sn RV) and grouper nervous necrosis virus (GNNV) co-infection of GF-1 cells showed that Sn RV enhanced the infectivity and CPE of GNNV [13], suggesting a complicated co-infection mechanism driven by interaction between the two viruses.

Viruses in the *Almendravirus* genus encode the N, P, M, G, and L proteins. Between the G and L proteins, they also encodes a small hydrophobin (U1) [7]. The conservation of amino acids in the coding region of the rhabdovirus L gene is higher than that of the other four proteins (N, P, M, and G) [6]; RdRp in the L gene coding region is a core protein that determines viral genome replication [7]. Recent studies have reported that amino acid identity of the coding region of the L protein of <90% is sufficient for identifying new rhabdovirus species [14].

As of 10 March 2021, GenBank contained the genome sequences of 13 *Almendravirus* species (Table S5), including the 2 rhabdoviruses isolated in this study; a total of 15 *Almendravirus* viruses have been sequenced. Our amino acid identity analysis of the L protein coding region of these 15 virus isolates showed 95.9–100% identity among the seven Menghai rhabdoviruses isolated in Yunnan and Guangdong Provinces in China, 99.6% identity between the two Balsa viruses isolated from Peru, and 90.7% identity between SXABTV isolated in this study and the ABTV isolated from Peru. Thus, these 11 virus isolates belonged to three *Almendravirus* species (Figure 4), among which 7 isolates belonged to Menghai rhabdovirus virus and 2 to Balsa virus. The nucleotide and amino acid lengths of the coding regions of the six genes from SXABTV isolated in this study were identical to those of ABTV; SXABTV had only one more amino acid T than ABTV, at the 188–190 position of the P gene. The amino acids of the L protein coding region had 90.7% identity between SXABTV and ABTV, indicating that SXABTV was an isolated strain of ABTV, which is found in different regions and different mosquito species.

SXARV isolated in this study had a full-length genome of 9590 nt, whereas that of SXABTV was 11,480 nt, and the nucleotides and amino acids of the coding regions of the six protein genes differed significantly between the two virus isolates (Table 1), with only 45.5% amino acid identity in the L gene coding region between the two viruses. The SXARV and SXABTV isolates obtained in this study clearly belong to two completely different virus species. Interestingly, although the results of our phylogenetic analysis showed that SXARV and the Rio Chico virus isolated from the Panamanian mosquito were of the same evolutionary branch, the total length of the SXARV isolate was different from known *Almendravirus* viruses, including Rio Chico virus. All *Almendravirus* viruses except SXARV had a genome length between 10,735 nt (Rio Chico virus) and 11,876 nt (Puerto Almendras virus) (Table 1), whereas the full length of the SXARV genome was only 9590 nt, representing the shortest genome among known *Almendravirus* viruses. The difference in length between SXARV and other *Almendravirus* viruses is mainly caused by a substantially shorter N protein encoded by SXARV. The highest identity of the L gene of SXARV was 63.3%, with a Rio Chico virus isolate. Since this value is <90%, SXARV can be considered a new rhabdovirus species [14]. In summary, SXARV is a novel virus species in the *Almendravirus* genus (*Rhabdoviridae*).

## 4. Materials and Methods

### 4.1. Specimen Collection

From June to August in 2019, we collected blood-sucking insects overnight (18:00 p.m. to 6:00 a.m.) at animal pens including chicken pens, dog houses, and mule pens in Wuxiang County, Shanxi Province, China (36°39′–37°8′ N, 112°26′–113°22′ E) using a blood-sucking insect collection tool (model no.: MM200BL; Guangzhou Changsheng Chemical Technology Service Co., Ltd., Guangzhou, China, https://guangzhou.11467.com/info/8049209.htm (accessed on 5 June 2019)) comprising a 12-V, 12-Ah battery, 0.1-A bulb, and 0.14-A fan. All mosquito specimens were placed in a −40 °C low-temperature refrigerator for 30 min and then removed for processing in an ice bath. Each specimen was labeled according to morphology, collection time, collection environment, and then stored in liquid nitrogen until laboratory testing.

### 4.2. Cell Lines

Golden hamster kidney (BHK-21) cells were cultured in 90% Eagle medium (laboratory preparation) containing 7% fetal bovine serum (FBS; Invitrogen, Carlsbad, CA, USA), 1% penicillin and streptomycin (100 U/mL), 1% glutamine (30 g/L), and 1% NaHCO_3_ in a 37 °C incubator with 5% CO_2_. Aedes albopictus egg (C6/36) cells were cultured in 89% RPMI 1640 (Invitrogen), 10% FBS (Invitrogen), and 1% penicillin and streptomycin (100 U/mL) in a 28 °C incubator [15,16,17].

### 4.3. Virus Isolation

To isolate viruses from mosquitoes, we pooled groups of 30–50 individuals according to collection site and species, put them into a glass grinder, and washed them twice with grinding solution consisting of 93% Eagle medium, 5% penicillin and streptomycin (100 U/mL), 1% glutamine (30 g/L), and 1% NaHCO_3_. We then added 1.5 mL grinding solution and ground the pooled sample in an ice bath. After centrifugation at 4 °C and 12,000 rpm for 30 min, 70 μL of ground supernatant was inoculated into 80% monolayer BHK-21 and C6/36 cells in a 24-well plate (Corning Inc., Corning, NY, USA). BHK-21 and C6/36 cells were continuously cultured in an incubator at 37 °C with 5% CO_2_ and at 28 °C, respectively. Cytopathic effect (CPE) was monitored under a microscope every 12 h. When CPE appeared, the virus solution was collected and stored in the refrigerator at −80 °C until identification. First-generation cell culture supernatant that did not show CPE was blind passaged for three generations and discarded if CPE did not appear after that [15,16,17].

### 4.4. Virus Plaque

C6/36 cells were transferred into 6-well culture plates (Corning Inc., NY, USA) to grow into monolayer cells with a coverage rate of 80%. Virus dilutions (10^–1^–10^–6^) were sequentially added to the 6-well culture plate (0.1 mL/well). The wells were placed in a 28 °C incubator for 1 h, and 1% agarose-MEM medium containing 2% FBS was added to each well to cover the cells (3 mL/well). After culturing for 5 days, a second layer of 1% agarose-MEM medium containing 7% neutral red and 2% FBS was spread over the cells (3 mL/well). The time and number of instances of plaque appearance were observed, and the plaque diameter was measured as previously described [18].

### 4.5. Electron Microscopy

When CPE reached 75%, virus-infected C6/36 cells were collected by centrifugation, and the sample was fixed with 2% formaldehyde and 2.5% glutaraldehyde solution. Following stepwise dehydration, soaking, embedding, polymerization, and trimming, 80 nm sections were prepared using an ultra-thin slicer and dried at room temperature for staining. The sections were stained with 1% uranyl acetate for 10 min and cleaned with ultrapure water. Next, the slices were stained with 0.02% lead citrate for 5 min and then cleaned and dried at room temperature for inspection. Observations were conducted using a transmission electron microscope (model no.: TF20; FEI Co., Hillsboro, OR, USA) [19].

### 4.6. Viral RNA Extraction and cDNA Library Preparation

Total RNA was extracted from the supernatant of virus-infected cells using the QIAamp Viral RNA Mini Kit (Qiagen, Valencia, CA, USA) according to the manufacturer’s instructions. The extracted RNA was placed in a 65 °C water bath for 10 min and then immediately placed in an ice bath for 2 min. Next, 32 μL RNA was added to the first-strand reaction tube of the Ready-To-Go kit (GE Healthcare, Little Chalfont, Buckinghamshire, UK), which was maintained at room temperature for 1 min before the addition of 1 μL pd(N)6 random primer (TaKaRa, Kyoto, Japan). This mixture was centrifuged immediately and incubated in a water bath at 37 °C for 1 h. The total volume of the cDNA library was 33 μL; it was stored at −40 °C until later use [19,20].

### 4.7. Virus Identification Using Total RNA Sequencing

To examine the viral genome of the SX1916 isolate, we extracted the total RNA of the supernatant of C6/36 cells infected with the SX1916 isolate for next-generation sequencing (NGS) using the QIAamp Viral RNA Mini Kit (QIAamp, Qiagen, Valencia, CA, USA). We used the TruSeq total RNA library kit to construct the cDNA library, and sequencing was performed on the HiSeq4000 platform (Illumina, San Diego, CA, USA). After low-quality reads were removed from the sequencing data, we performed de-novo assembly. We extracted virus-related contigs based on the taxonomic information of the blast hits. To avoid mis-assembly, reads were mapped back to the virus genome with Bowtie2 (version 2.3.5.1, Ben Langmead) and inspected with Geneious Program (version 9.1.5, Auckland, New Zealand) [21]. The viral genome sequences obtained by NGS was used as template to design overlapping PCR primers for sequence confirmation.

### 4.8. Viral Gene Amplification and Nucleotide Sequencing

We applied polymerase chain reaction (PCR) for viral gene amplification in a 25 μL system comprising 2 μL cDNA as template, 12.5 μL 2× GoTaq Green Master Mix, 8.5 μL nuclease-free water, and 1 μL each of 10 pmol/μL upstream and downstream primers. After the PCR was complete, 5 μL of gene amplification products was detected by 1% agarose gel electrophoresis [19,20]. The primers used in this study are listed in Tables S2 and S3. The positive PCR products were sequenced following the four-color fluorescent labeling dideoxy termination method (Tianyi Huiyuan Biotechnology Co., Ltd., Beijing, China).

### 4.9. Nucleotide Sequence Analysis

The nucleotide sequence was compared to non-redundant protein (nr) database available at the National Center for Biotechnology Information (NCBI) website (https://blast.ncbi.nlm.nih.gov/Blast.cgi (accessed on 5 May 2021)) using blastn program. We used the Seqman software (DNAStar, Madison, WI, USA) for nucleotide sequence splicing and quality analysis and the BioEdit software (Version 7.0, Tom Hall) for multiple sequence alignment. We used the MEGA software (Version 7.0, Kumar, Stecher, and Tamura 2015) to complete the systematic evolution analysis based on the maximum likelihood method, with a bootstrap value of 1000; the General Time Reversible model is used in analysis. We used the MegAlign (Madison, WI, USA) tool for identity analysis of nucleotide and amino acid sequences [16,19]. Strain information used in virus molecular genetic evolution analysis is provided in Table S5.

## 5. Conclusions

In the study, two rhabdoviruses, SXARV and SXABTV, were found in a pool of *Armigeres subalbatus* samples collected in Shanxi Province, China. Amino acid identity analysis shows that SXARV is a novel virus species in the *Almendravirus* genus (*Rhabdoviridae*). Despite the isolation of a variety of *Almendravirus* viruses in Asia, North America, and South America, it remains unclear whether these viruses could infect mammalian hosts. Therefore, further investigations of the infection status of *Almendravirus* in domestic and wild animals will be of great importance.

## Figures and Tables

**Table:** Subtable A

Virus	Country	Host	Year	The Total Length	N		P		M	
					nt (%)	aa (%)	nt (%)	aa (%)	nt (%)	aa (%)
SXARV	China	*Armigeres subalbatus*	2019	9590	471	156	636	211	480	159
SXABTV	China	*Armigeres subalbatus*	2019	11,480	1305 (-/49.7%)	434 (-/29.8%)	840 (-/39.5%)	279 (-/20.3%)	480 (-/34.3%)	159 (-/13.5%)
Arboretum virus	Peru	Psorophora albigenu mosquitoes	2009	11,482	1305 (83.9%/48.2%) *	434 (97.5%/30.4%) **	843 (70.8%/39.1%)	280 (68.5%/19%)	480 (79.3%/36.1%)	159 (88.5%/15.4%)
Puerto Almendras virus	Peru	Ochlerotatus fulvus	2009	11,876	1305 (61.2%/44.3%)	434 (59.6%/28%)	963 (43.2%/35%)	320 (22.3%/8.5%)	480 (58.7%/33.9%)	159 (48.1%/17.3%)
Balsa virus (CoB 76)	Colombia	Psorophora albigenu	2013	11,287	1296 (47.4%/54.8%)	431 (32.3%/38.5%)	768 (38.9%/46.4%)	255 (23.3%/28.9%)	549 (35.7%/39.8%)	182 (14.7%/26.9%)
Balsa virus (CoB 84)	Colombia	Psorophora albigenu	2013	11,286	1296 (47.4%/54.8%)	431 (32.3%/38.5%)	768 (39.1%/46.2%)	255 (23.3%/28.9%)	549 (35.5%/39.8%)	182 (14.7%/26.9%)
Coot Bay virus	USA	Anopheles quadrimaculatus	2013	10,869	1290 (41.9%/45.6%)	429 (25.5%/29.8%)	696 (34.5%/37.2%)	231 (18.7%/29.5%)	528 (40.9%/35.9%)	175 (14.7%/19.9%)
Menghai rhabdovirus (Menghai )	China	Aedes albopictus	2010	10,744	1278 (43.6%/45.4%)	425 (28%/32.9%)	777 (35.5%/32.8%)	258 (18.4%/23.9%)	501 (40%/34.1%)	166 (12.2%/21.8%)
Menghai rhabdovirus (kunoichi)	Japan	Aedes albopictus cultured cell line C6/36	2018	10,777	1278 (43.8%/45.4%)	425 (28%/32.9%)	777 (35.5%/32.8%)	258 (18.4%/23.9%)	501 (41.3%/33.7%)	166 (12.2%/20.5%)
Menghai rhabdovirus (GD18003)	China	Midges	2018	10,744	1278 (42.5%/44.8%)	425 (28%/32.9%)	777 (35.8%/33.8%)	258 (18.4%/23.9%)	501 (40.9%/33.3%)	166 (12.2%/21.2%)
Menghai rhabdovirus (GD18004)	China	Midges	2018	10,743	1278 (42.5%/44.8%)	425 (28%/32.9%)	777 (35.6%/33.8%)	258 (18.4%/23.9%)	501 (40.9%/33.3%)	166 (12.2%/21.2%)
Menghai rhabdovirus (GD18005)	China	Midges	2018	10,744	1278 (42.5%/44.8%)	425 (28%/32.9%)	777 (35.8%/33.8%)	258 (18.4%/23.9%)	501 (40.9%/33.3%)	166 (12.2%/21.2%)
Menghai rhabdovirus (GD18008)	China	Midges	2018	10,744	1278 (42.5%/44.8%)	425 (28%/32.9%)	777 (35.6%/33.8%)	258 (18.4%/23.9%)	501 (40.9%/33.3%)	166 (12.2%/21.2%)
Menghai rhabdovirus (GD18010)	China	Midges	2018	10,744	1278 (42.9%/45.6%)	425 (28%/32.9%)	777 (34.8%/33.2%)	258 (18.4%/23.9%)	501 (40.5%/33.7%)	166 (12.2%/21.2%)
Rio Chico virus	Panama	Anopheles triannulatus	2013	10,735	1317 (42.5%/59%)	438 (26.7%/52.8%)	642 (41.6%/55%)	213 (21.%/54.8%)	480 (34.9%/59.5%)	159 (17.3%/48.1%)

**Table:** Subtable B

Virus	G		U1		L	
	nt (%)	aa (%)	nt (%)	aa (%)	nt (%)	aa (%)
SXARV	1350 (/-)	449 (/-)	162 (/-)	53 (/-)	6189 (/-)	2062 (/-)
SXABTV	1431 (-/38.7%)	476 (-/22.2%)	222 (-/41.4%)	73 (-/29.4%)	6189 (-/54.3%)	2062 (-/45.5%)
Arboretum virus	1431 (75.6%/40.1%)	476 (84.1%/22%)	222 (69.4%/37.7%)	73 (52.9%/27.1%)	6189 (78.9%/54.8%)	2062 (90.7%/45.4%)
Puerto Almendras virus	1413 (52.4%/39.8%)	470 (35.6%/24.4%)	243 (42.9%/30.6%)	80 (28.2%/16.5%)	6180 (65.3%/54.3%)	2059 (64.1%/46.3%)
Balsa virus (CoB 76)	1356 (39.9%/48.8%)	451 (19.7%/27.8%)	219 (55.6%/40.3%)	72 (24.7%/21.2%)	6162 (55.4%/59.2)	2053 (44%/52.7)
Balsa virus (CoB 84)	1356 (40%/48.7%)	451 (19.7%/27.8%)	219 (56.3%/41%)	72 (25.9%/21.2%)	6162 (55.3%/59.2)	2053 (44%/52.7)
Coot Bay virus	1329 (40.3%/43.9%)	442 (19.7%/29%)	180 (39.2%/57.1%)	59 (27.1%/35.3%)	6108 (51.9%/54.3%)	2035 (41%/44.4%)
Menghai rhabdovirus (Menghai)	1332 (38.7%/42.9%)	443 (20.6%/29.2%)	123 (36.6%/53%)	40 (21.2%/23.5%)	6099 (51.4%/53.2%)	2032 (40.3%/44.4%)
Menghai rhabdovirus (kunoichi)	1356 (38.6%/42%)	451 (20.6%/29.4%)	210 (24.6%/35.1%)	69 (25.9%/27.1%)	6099 (51.3%/53%)	2032 (40.0%/44%)
Menghai rhabdovirus (GD18003)	1332 (39.1%/43%)	443 (21.4%/29.7%)	123 (36.6%/53%)	40 (20%/23.5%)	6099 (51.2%/53.2%)	2032 (40.0%/44.3%)
Menghai rhabdovirus (GD18004)	1332 (39.1%/43%)	443 (21.4%/29.7%)	123 (36.6%/53%)	40 (20%/23.5%)	6099 (51.4%/53.4%)	2032 (40.3%/44.5%)
Menghai rhabdovirus (GD18005)	1332 (39.1%/43%)	443 (21.4%/29.7%)	123 (36.6%/53%)	40 (20%/23.5%)	6099 (51.2%/53.2%)	2032 (40.0%/44.3%)
Menghai rhabdovirus (GD18008)	1332 (39.1%/43%)	443 (21.4%/29.7%)	123 (36.2%/52.6%)	40 (21.2%/23.5%)	6099 (51.5%/53.5%)	2032 (40.1%/44.4%)
Menghai rhabdovirus (GD18010)	1332 (38.3%/42.6%)	443 (20.1%/28.6%)	123 (35.8%/53%)	40 (21.2%/23.5%)	6099 (51.4%/53.5%)	2032 (40.2%/44.3%)
Rio Chico virus	1344 (40.4%/53.4%)	447 (22.7%/41.7%)	156 (42.9%/57.5%)	51 (23.5%/49.4%)	6189 (54.3%/64.6%)	2062 (45.2%/63.3%)

Note: (1) The adjacent gray-colored strains are different isolates of the same virus (2 strains of Arboretum virus, 2 strains of Balsa virus, 7 strains of Menghai rhabdovirus). (2) 1305 (83.9%/48.2%) * denotes the length of N protein nucleotide sequence of arboretum virus (nucleotide identity between N protein of Arboretum virus and SXABTV/nucleotide identity between N protein of Arboretum virus and SXARV); (3) 434 (97.5%/30.4%) ** represented the amino acid length of the N protein of Arboretum virus (amino acid identity between N protein of Arboretum virus and SXABTV/amino acid identity between N protein of Arboretum virus and SXARV); (4) 40–64.1% indicated the lowest and highest values of amino acid identity in the coding region of the L gene of *Almendravirus* Genus, respectively. (5) Alignment of Amino acid sequence of SXABTV and SXARV is shown in Figure S4. (6) Subtable A followed by subtable B.

## Data Availability

Sequences were submitted to GenBank with accession numbers MW890015 and MW890016.

## Supplementary Materials

The following are available online at https://www.mdpi.com/article/10.3390/pathogens11060624/s1, Table S1: Summary of mosquito specimens collected from June to August 2019 in Wuxiang County, Shanxi Province; Table S2: Viral gene amplification primers used for initial virus identification in this study; Table S3: Taxonomy annotation of all virus contigs. Table S4: Shanxi Armigeres subalbatus rhabdovirus (SXARV) and Shanxi Arboretum virus (SXABTV) genome nucleotide sequence amplification primers used in this study; Table S5: Virus strains used in virus molecular genetic evolution analysis in this study; Figure S1: Plaque purification of the SX1916 virus isolate; Figure S2: SXARV and SXABTV gene amplification in fourth-generation C6/36 cells inoculated with the SX1916 virus isolate; Figure S3. Electron microscopic morphology of SXARV. Figure S4: Alignment of Amino acid sequence of the Shanxi Arboretum virus (SXABTV) and Shanxi Armigeres subalbatus rhabdovirus (SXARV). Figure S5. SX1916 virus (SXABTV and SXARV) and SXARV gene amplification.
