# Supplementary material for: Two Rhabdoviruses, One Novel, Isolated from Armigeres subalbatus in China
Source: Pathogens. 2022 May 27;11(6):624. doi: 10.3390/pathogens11060624 (PMC9230728; doi:10.3390/pathogens11060624)
Supplement: Supplementary file 1 [file pathogens-11-00624-s001.zip › pathogens-1679819-supplementary.pdf]

Table S1. Summary of mosquito specimens collected from June to August 2019 in Wuxiang County, Shanxi Province.

The collection site	Anopheles sinensis	Armigeres subalbatus	Aedimorphus vexans	Culex pipiens pallens	The total
Collection site 1	326	248	2	2	578
Collection site 2	0	17	7	0	24
Collection site 3	14	92	28	43	177
Collection site 4	0	3	0	0	3
The total	340(43.48%)	360(46.04%)	37(4.73%)	45(5.75%)	782
The pools	7	7	1	1	16

Note:Collection site 1 was a mule pen, collection site 2 comprised mule and chicken pens, collection site 3 comprised a mule pen and dog kennel, from where SX1916 isolate was obtained, and collection site 4 was a chicken pen.

Table S2. Viral gene amplification primers used for initial virus identification in this study.

primer	The amplified segment	Sequence of primers(5'-3')	The length of amplification（bp）
Flavivirus			
FU1	NS5	TAC AAC ATG ATG GGA AAG AGA GAG AA	266bp
cFD2		GTG TCC CAG CCG GCG GTG TCA TCA GC	
Alphavirus			
M2W(+)	NSP1	YAG AGC DTT TTC GCA YST RGC HW	434bp
cM3W		ACA TRA ANK GNG TNG TRT CRA ANC CDA YCC	
M2W2(+)		TGY CCN VTG MDN WSY VCN GAR GAY CC	310bp
Bunyavirus			
BUP	S	ATG ACT GAG TTG GAG TTT GAT GTC GC	251bp
BDW		TGT TCC TGT TGC CAG GAA AAT	
Banna virus			
BAV-12-854-S	12S	AAA TTG ATA GYG YTT GCG TAA GAC	850bp
BAV-12-B2-R		GTT CTA AAT TGG ATA CGG CGT GC	
OYA virus			
OYA-11F	NS	GGT TAA TAA CCA TTT TCC CCA	310bp
OYA-2R		ACC TTC CTC ATG AAG TTG ACA	
Tibet orbivirus			
6-4-2F	4s	CGA CAG ACC AAA AGA TAT	848bp
6-4-2R		TCA ACA CGT AAT CCA ATA	
Japanese encephalitis virus			
JE-955F	E	TGYTGG TCG CTC CGG CTT A	1581bp
JE-2536R		AAG ATG CCA CTT CCA CAY CTC	
Densovirus			
DNV-F	NS1	AACCGTTGGTGACCTCTACCCAC	1105bp
DNV-R		GATTTTCCCATTGCTGTGCCGTTG	
Totivirus			
Toti2-947F		GCAATGGAGAACAAATTCACG	825bp
Toti2-1771R		CTGGTGTGCTTGTTTCTGTTTC	

Table S3: Taxonomy annotation of all virus contigs.

No.	Length	Accession	Blastx	Identity	E value	Order	Family	Subfamily	Genus	Species
Contig1	522	YP_009094378.1	YP_009094378.1 N protein [Arboretum almendravirus]	76.2	2.90E -26	Mononegavir ales	Rhabdovi ridae	Alpharhab dovirinae	Almendravirus	Arboretum almendravirus
Contig2	525	YP_009094379.1	YP_009094379.1 P protein [Arboretum almendravirus]	89.4	4.80E -13	Mononegavir ales	Rhabdovi ridae	Alpharhab dovirinae	Almendravirus	Arboretum almendravirus
Contig3	11704	YP_009094383.1	YP_009094383.1 L protein [Arboretum almendravirus]	90.6	0	Mononegavir ales	Rhabdovi ridae	Alpharhab dovirinae	Almendravirus	Arboretum almendravirus
Contig4	423	QEM39328.1	QEM39328.1 NP, partial [Guadeloupe mosquito quaranja-like virus 3]	26.8	6.60E -05	Articulavirale s	Orthomy xoviridae	NA	Quaranjavirus	Guadeloupe mosquito quaranja-like virus 3
Contig5	446	YP_009094383.1	YP_009094383.1 L protein [Arboretum almendravirus]	95.7	3.00E -16	Mononegavir ales	Rhabdovi ridae	Alpharhab dovirinae	Almendravirus	Arboretum almendravirus
Contig6	937	YP_009094378.1	YP_009094378.1 N protein [Arboretum almendravirus]	80.4	2.50E -12	Mononegavir ales	Rhabdovi ridae	Alpharhab dovirinae	Almendravirus	Arboretum almendravirus
Contig7	9726	YP_009094394.1	YP_009094394.1 L protein [Puerto Almendras virus]	47.1	0	Mononegavir ales	Rhabdovi ridae	Alpharhab dovirinae	Almendravirus	Puerto Almendras almendravirus
Contig8	463	YP_009094378.1	YP_009094378.1 N protein [Arboretum almendravirus]	90.3	4.90E -38	Mononegavir ales	Rhabdovi ridae	Alpharhab dovirinae	Almendravirus	Arboretum almendravirus
Contig9	474	ANW72245.1	ANW72245.1 putative glycoprotein, partial [Guato virus]	98.1	2.60E -82	NA	NA	NA	NA	Guato virus

Table S4. Shanxi *Armigeres subalbatus* rhabdovirus (SXARV) and Shanxi Arboretum virus (SXABTV) genome nucleotide sequence amplification primers used in this study.

primer	The amplified segment	Sequence of primers(5’-3’)	The length of amplification（bp）
SXABTV			
AB1-1F	N	CCCCTTATAAAGCACACGCC	437bp
AB1-437R		ACATTGATGGATCATCGGGC	
AB2-121F	N	TGCCCTAGTTCTCATGTTCCC	977bp
AB2-1097R		ACTCTGCATCTGTCATCGAGAA	
AB3-849F	N、P	CCACATTGTTGATGGGCGAA	1205bp
AB3-2053R		TGATCTATGGCTAGAGCTTGGT	
AB4-1883F	P、M	GACGAGATCATGACTCCCAACA	1171bp
AB4-3053R		CGAACGAGCCTACTTCTGGG	
AB5-2901F	M、G	TCCTGTTGACGAAACGTGGA	1209bp
AB5-4109R		TGGCATTCCTAAGGGAGGGT	
AB6-3882F	G	ACTGCCCGAAAGTTGAGGAG	1154bp
AB6-5035R		AGATACCGTAGACCTGTCGT	
AB7-4705F	U1、L	AACCAAGGGTCACGAATTGGA	1415bp
AB7-6119R		ATCAGCGATCTTTCCCACCA	
AB8-5908F	L	GGAGGTACTCTAAAGACTTGCCTG	1273bp
AB8-7180R		ATTCCTTCAAAGCCCCCTGG	
AB9-6997F	L	AAGCTGTCGGACCGGTTTTT	1155bp
AB9-8151R		ATCGAGGGAAGCAGGGTTTT	
AB10-7811F	L	GTTCCCGGATCCTATAACCGA	1145bp
AB10-8955R		GACTTGCACCTATGGTTTCTGC	
AB11-8763F	L	AAGAGACGCTCAGGAACTGC	985bp
AB11-9747R		TCCTGATACGTGATGTGTTCGG	
AB12-9516F	L	CCACCAGAATATCCTCCGAGT	1289bp
AB12-10804R		TCACGAAGAGACGGTGGAATC	
AB13-10700F	L	AAGCTGTCGGACCGGTTTTT	900
AB13-11600R		ATCGAGGGAAGCAGGGTTTT	

SXARV			
AR1-1F	N	AATGGGTTTAATTACATGAGTC	425bp
AR1-425R		TTGACTCTCCCTTTAGCCCAT	
AR2-303F	N、P	TGCCATGGGTAGCGTAGAAG	744bp
AR2-1046R		CCTGGTTTGTCAACTCCGGT	
AR3-911F	P、M	CCGGATGAAGTTTTGGTGCC	855bp
AR3-1765R		GCTTCGATCAAGTTGCACGG	
AR4-1626F	G	ACGGGCCAGAGTTTAAAGGAT	1031bp
AR4-2656R		AGTGGGGTGCAGCTTCCTAA	
AR5-2329F	G、U1、L	TGTCAATACCATGTATGTAGCGGA	1405bp
AR5-3733R		AGTGTGAGCAGTAATAAACTCTTCT	
AR6-3513F	L	TCAGCTTGAACTCCCCGATT	1221bp
AR6-4733R		CCCATCTACCCTCTAATTGGTCA	
AR7-4597F	L	AATGAGACACTTCCACACAACT	849bp
AR7-5445R		ACTAATGACCACCCCTTTTGTCT	
AR8-5242F	L	AGATGAGGCAGTAGGTCCAGT	948bp
AR8-6189R		TTGTTCAATGAAGTCGCAGTGA	
AR9-6046F	L	AAGGCGATTTCCAGACCCAA	992bp
AR9-7037R		ATCTGTGTTGCGCTGTACCA	
AR10-6831F	L	GTGCATTACAGCCATGGGAAA	1284bp
AR10-8114R		GCAGTTCTTGAGAGCTGACTTG	
AR11-7803F	L	CCTCCTTGGCAGTTAGTGGA	1186bp
AR11-8988R		GCCCTAGCAAATTCCTCTACATC	
AR12-8707F	L	TGCTGCTAACTTCATCAAAGGC	1018bp
AR12-9724R		CAGACGTGTGCTCTTCCGAT	

Table S5. Virus strains used in virus molecular genetic evolution analysis in this study.

No.	Genus	Virus	Year	Country	Host	Isolate	Genbank number
1	Almendravirus	Shanxi Arboretum virus	2019	China	Armigeres subalbatus	SXABTV1916-1	MW890015
2		Shanxi Armigeres subalbatus rhabdovirus	2019	China	Armigeres subalbatus	SXARV1916-2	MW890016
3		Arboretum virus	2009	Peru	Psorophora albigenu mosquitoes	Lo-121	NC_025393.1
4		Balsa virus	2013	Colombia	Psorophora albigenu	CoB 76	NC_039200.1
5		Balsa virus	2013	Colombia	Psorophora albigenu	CoB 84	KX228199
6		Coot Bay virus	2013	USA	Anopheles quadrimaculatus	EVG5-53	NC_031957.1
7		Menghai rhabdovirus	2010	China	Aedes albopictus	Menghai	NC_040602.1
8		Menghai rhabdovirus	2018	Japan	Aedes albopictus cultured cell line C6/36	kunoichi	LC270812
9		Menghai rhabdovirus	2018	China	Midges	GD18003	MT240824.1
10		Menghai rhabdovirus	2018	China	Midges	GD18004	MT240825.1
11		Menghai rhabdovirus	2018	China	Midges	GD18005	MT240826.1
12		Menghai rhabdovirus	2018	China	Midges	GD18008	MT240827.1
13		Menghai rhabdovirus	2018	China	Midges	GD18010	MT240828.1
14		Puerto Almendras virus	2009	Peru	Ochlerotatus fulvus	LO-39	NC_025395.1
15		Rio Chico virus	2013	Panama	Anopheles triannulatus	GAM 195	NC_031958.1
16	Alphanemrhavirus	Xingshan nematode virus	2014	China	Spirurian nematodes	XSNXC32924	NC_033701.1
17	Alphanucleorhabdovirus	Potato yellow dwarf virus	2008	USA	/	SYDV	NC_016136.1
18	Arurhavirus	Aruac virus	1955	Trinidad and Tobago: Trinidad	Trichoprosopon theobaldi	TRVL9223	KM204987.1
19	Barhavirus	Bahia grande virus	1974	USA	Ochlerotatus sollicitans	TB4-1054	KM205018.1
20	Betanucleorhabdovirus	Sonchus yellow net virus	1992	/	Nicotiana edwardsonii	/	L32603.1
21	Caligrhavirus	Lepeophtheirus salmonis rhabdovirus	2013	Norway	Lepeophtheirus salmonis	LSRV-No127	KJ958536.1
22	Curiovirus	Curionopolis virus	1985	Brazil	Culicoides sp.	BeAr440009	NC_039201.1

23	Cytorhabdovirus	Lettuce necrotic yellows virus	/	Australia	Allium sativum (garlic)	318	NC_007642.1
24	Dichorhavirus	Citrus chlorotic spot virus	2017	Brazil	Talipariti tiliaceum (L.) Fryxell (syn. Hibiscus tiliaceus L.	Trs3	MG970599.1
25	Ephemerovirus	Bovine ephemeral fever virus	1968	/	Cow	BB7721	NC_002526.1
26	Gammanucleorhabdovirus	Maize fine streak virus	2004	USA	/	/	NC_005974.1
27	Hapavirus	Flanders virus	1961	USA	Culiseta melanura	61-7484	NC_039202.1
28	Ledantevirus	Le Dantec virus	1965	Senegal	Homo sapiens	DakHD763	NC_034443.1
29	Lostrhavirus	Lone star tick rhabdovirus	2009	USA	Amblyomma americanum	TickAa42	KU127239.1
30	Lyssavirus	Rabies virus	/	/	/	/	NC_001542.1
31	Mousrhavirus	Moussa virus	2004	Cote d'Ivoire	Culex decens	C23	NC_025359.1
32	Novirhabdovirus	Infectious hematopoietic necrosis virus (IHNV)			Oncorhynchus tschawytscha	WRAC	NC_001652.1
33	Ohlsrhavirus	Ohlsdorf virus	2012	Germany	Ochlerotatus cantans	Germany/2012/Oc.cantans	KY768856.1
34	Perhabdovirus	Perch rhabdovirus	1981	France	Perca fluviatilis	PRV	NC_020803.1
35	Sawgrhavirus	Sawgrass virus	1964	USA	Dermacentor variabilis	64A-1247	KM205013.1
36	Sigmavirus	Drosophila melanogaster sigmavirus	uncertain	France	Drosophila melanogaster	HAP23	NC_038281.1
37	Sprivivirus	spring viremia of carp virus	/	/	Cyprinus carpio	ATCC VR-1390	NC_002803.1
38	Sripuvirus	Niakha virus	1992	Senegal	Phlebotomus duboscq and Sergentomyia sp.	DakArD 88909	NC_025405.1
39	Sunrhavirus	Sunguru virus	2013	Uganda	chicken	Ug#41	NC_025401.1
40	Tibrovirus	Tibrogargan virus	1976	Australia	Culicoides brevitarsis	CS132	NC_020804.1
41	Tupavirus	Durham virus		USA	bird		NC_038284.1
42	Varicosavirus	Lettuce big-vein associated virus		/	/	/	NC_011558.1
43	Vesiculovirus	Vesicular stomatitis Indiana virus	1998	USA: Colorado	equine	98COE	NC_038236.1
44	Zarhavirus	Zahedan rhabdovirus	2001	Iran	Hyalomma anatolicum anatolicum	Ar Teh 157764	NC_040664.1

Note: / indicates that no relevant information was found in the National Center for Biotechnology Information (NCBI) database.

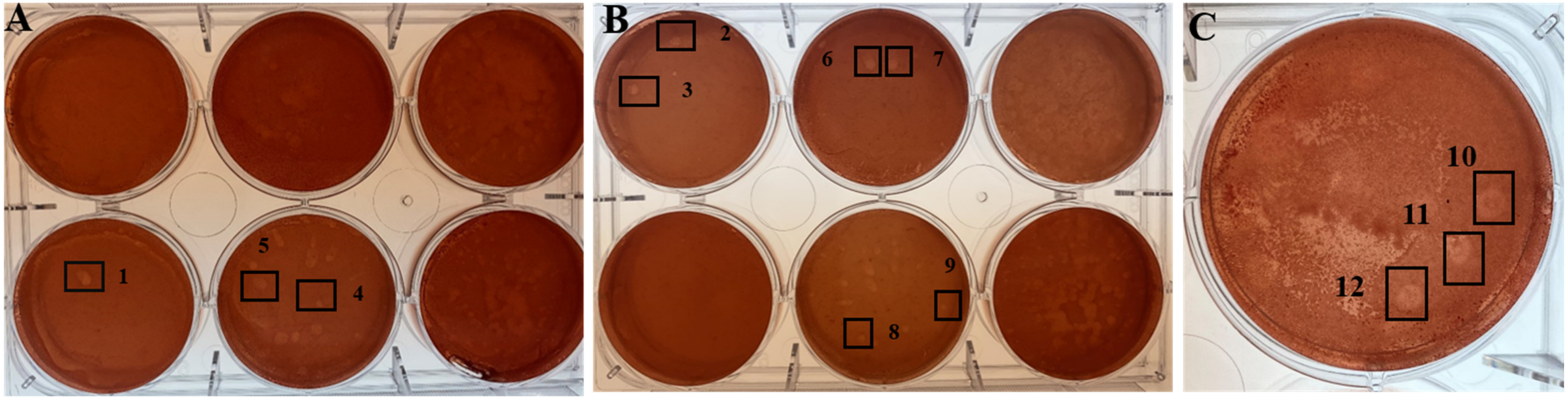

Figure S1. Plaque purification of the SX1916 virus isolate.

Note:A and B) Nine virus plaques were obtained in the first plaque purification assay. C) Three virus plaques were obtained in the second plaque purification assay.

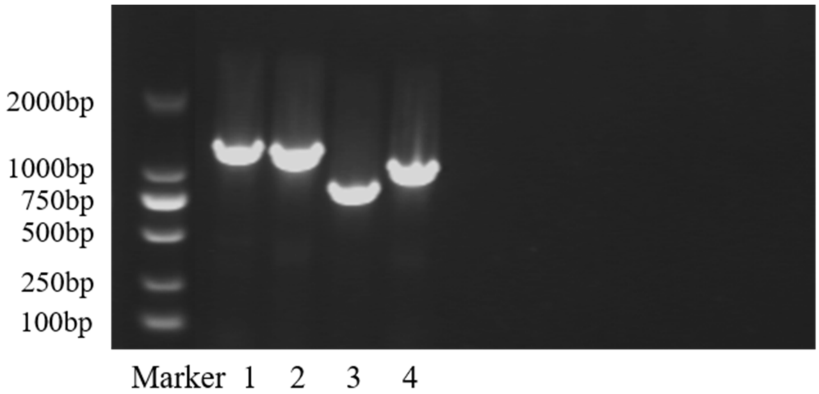

Figure S2. SXARV and SXABTV gene amplification in fourth-generation C6/36 cells inoculated with the SX1916 virus isolate. Note:The first and second channels in the electrophoresis diagram are positive amplification products of the SXABTV N and P genes, respectively. The third and fourth channels are positive amplification products of the SXARV P and M genes, respectively.

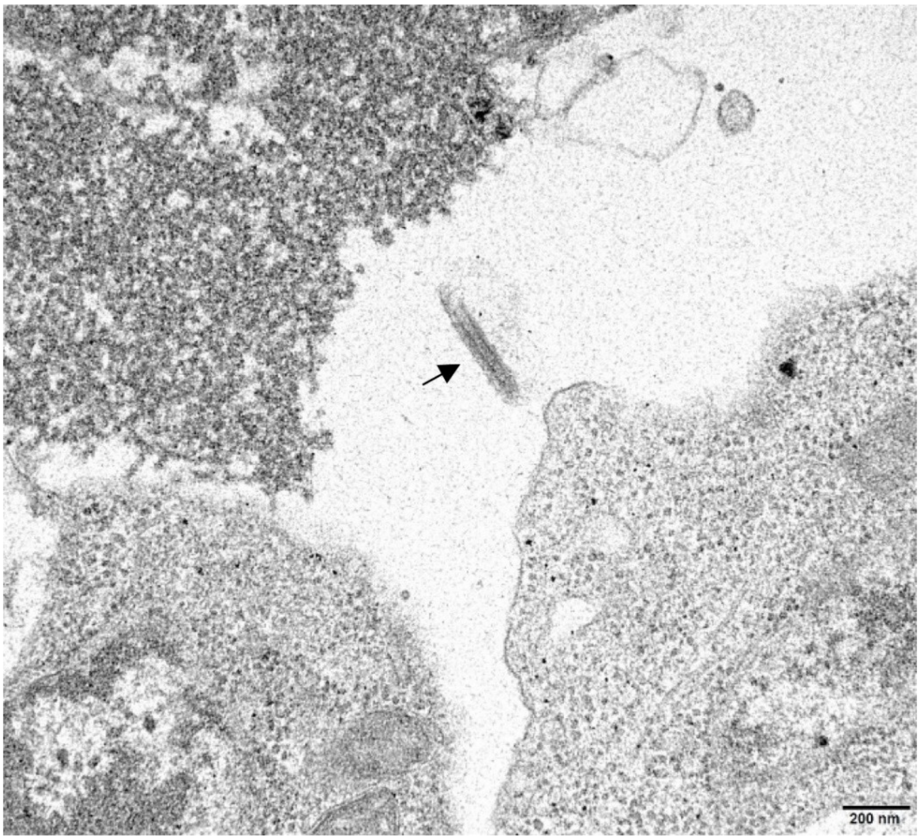

Figure S3. Electron microscopic morphology of SXARV.

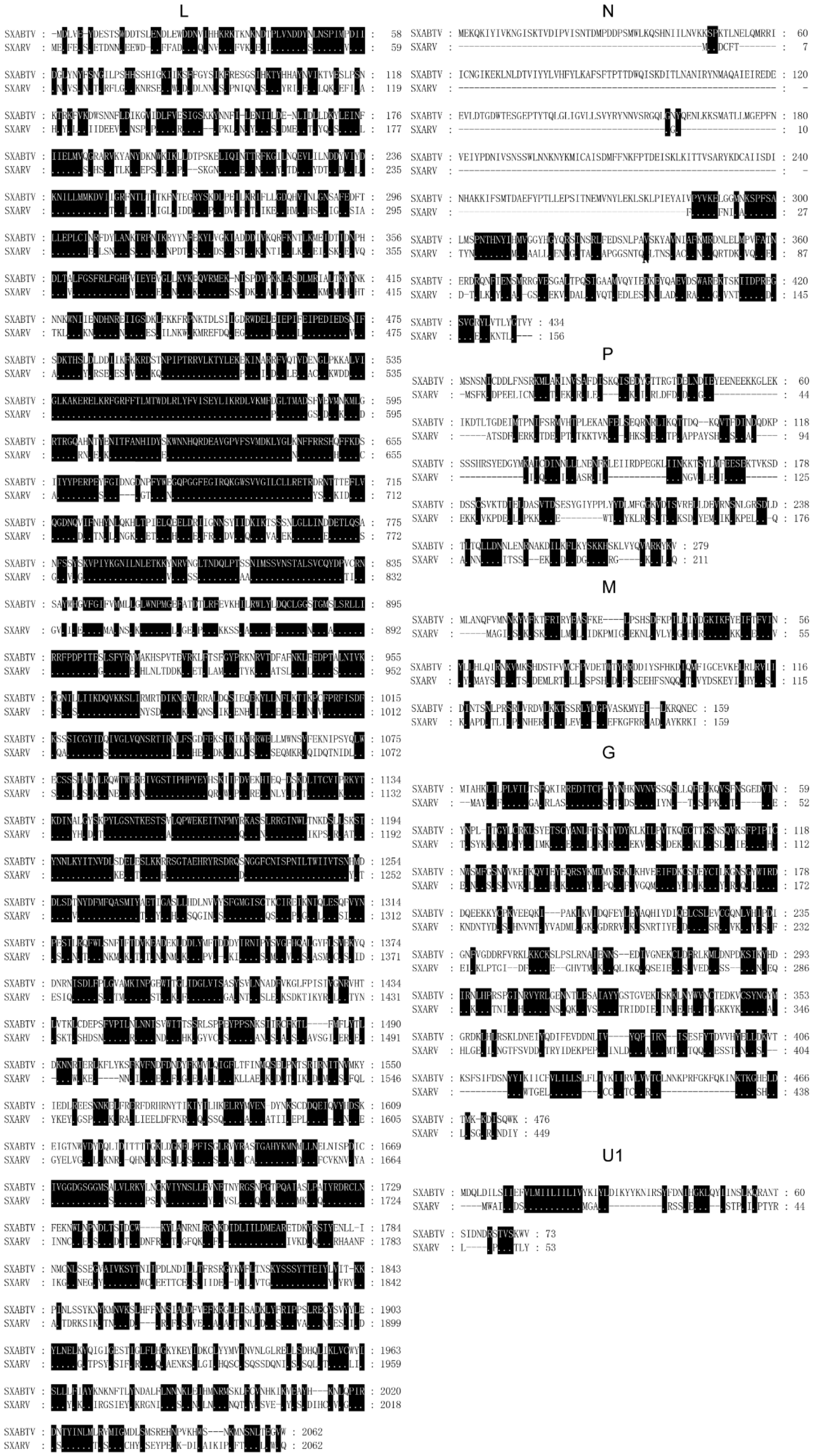

Figure S4. Alignment of Amino acid sequence of the Shanxi Arboretum virus (SXABTV) and Shanxi Armigeres subalbatus rhabdovirus (SXARV)

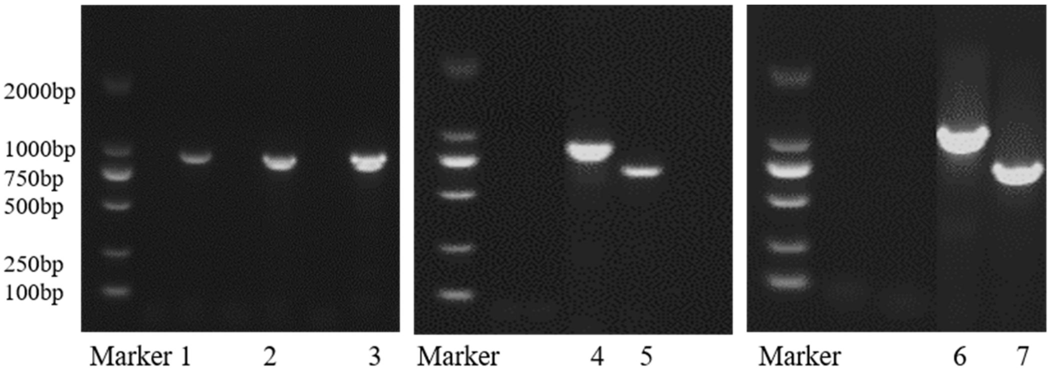

Figure S5. SX1916 virus(SXABTV and SXARV) and SXARV gene amplification

Note: The first channel, the second channel and the third channel in the electrophoresis diagram are positive amplification products of SXARV-infected c6/36 cells of 1-3 generations, respectively. The fourth and fifth channels were positive amplification products of SXABTV and SXARV infected second generation C6/36 cells, respectively. The sixth and seventh channels were positive amplification products of SXABTV and SXARV infected fourth generation C6/36 cells, respectively.
